# Timing of Antidepressant Discontinuation During Pregnancy and Postpartum Psychiatric Outcomes in Denmark and Norway

**DOI:** 10.1001/jamapsychiatry.2023.0041

**Published:** 2023-03-08

**Authors:** Nhung T. H. Trinh, Trine Munk-Olsen, Naomi R. Wray, Veerle Bergink, Hedvig M. E. Nordeng, Angela Lupattelli, Xiaoqin Liu

**Affiliations:** 1 PharmacoEpidemiology and Drug Safety Research Group, Department of Pharmacy, University of Oslo, Oslo, Norway; 2 NCRR–The National Centre for Register-based Research, Aarhus University, Aarhus, Denmark; 3 Department of Clinical Research, University of Southern Denmark, Odense, Denmark; 4 Institute for Molecular Bioscience, The University of Queensland, Brisbane, Queensland, Australia; 5 Queensland Brain Institute, The University of Queensland, Brisbane, Queensland, Australia; 6 Department of Psychiatry, Icahn School of Medicine at Mount Sinai, New York, New York; 7 Department of Psychiatry, Erasmus Medical Center, Rotterdam, the Netherlands; 8 Department of Child Health and Development, Norwegian Institute of Public Health, Oslo, Norway

## Abstract

**Importance:**

Approximately one-half of women treated for affective disorders discontinue antidepressant use during pregnancy, yet this discontinuation could lead to relapse post partum.

**Objective:**

To investigate the associations between longitudinal antidepressant fill trajectories during pregnancy and postpartum psychiatric outcomes.

**Design, Setting, and Participants:**

This cohort study used nationwide registers in Denmark and Norway. The sample included 41 475 live-born singleton pregnancies in Denmark (1997-2016) and 16 459 in Norway (2009-2018) for women who filled at least 1 antidepressant prescription within 6 months before pregnancy.

**Exposures:**

Antidepressant prescription fills were obtained from the prescription registers. Antidepressant treatment during pregnancy was modeled using the k-means longitudinal method.

**Main Outcomes and Measures:**

Initiation of psycholeptics, psychiatric emergencies, or records of self-harm within 1 year post partum. Between April 1 and October 30, 2022, hazard ratios (HRs) for each psychiatric outcome were estimated using Cox proportional hazards regression models. Inverse probability of treatment weighting was used to control for confounding. Country-specific HRs were pooled using random-effects meta-analytic models.

**Results:**

Among 57 934 pregnancies (mean [SD] maternal age, 30.7 [5.3] years in Denmark and 29.9 [5.5] years in Norway), 4 antidepressant fill trajectories were identified: early discontinuers (31.3% and 30.4% of the included pregnancies in Denmark and Norway, respectively), late discontinuers (previously stable users) (21.5% and 27.8%), late discontinuers (short-term users) (15.9% and 18.4%), and continuers (31.3% and 23.4%). Early discontinuers and late discontinuers (short-term users) had a lower probability of initiating psycholeptics and having postpartum psychiatric emergencies vs continuers. A moderately increased probability of initiation of psycholeptics was found among late discontinuers (previously stable users) vs continuers (HR, 1.13; 95% CI, 1.03-1.24). This increase in late discontinuers (previously stable users) was more pronounced among women with previous affective disorders (HR, 1.28; 95% CI, 1.12-1.46). No association between antidepressant fill trajectories and postpartum self-harm risk was found.

**Conclusions and Relevance:**

Based on pooled data from Denmark and Norway, a moderately elevated probability of initiation of psycholeptics in late discontinuers (previously stable users) vs continuers was found. These findings suggest that women with severe mental illness who are currently on stable treatment may benefit from continuing antidepressant treatment and personalized treatment counseling during pregnancy.

## Key Points

**Question:**

Are longitudinal antidepressant fill trajectories during pregnancy associated with postpartum psychiatric outcomes?

**Findings:**

In this cohort study of 57 934 pregnancies and 4 antidepressant fill trajectory groups in Denmark and Norway, early discontinuation and late discontinuation (short-term use) of antidepressants during pregnancy were associated with a decrease in initiating psycholeptics and having psychiatric emergencies post partum. Compared with continuing antidepressants during pregnancy, late discontinuation (previously stable use) was associated with an elevated moderate probability of postpartum psycholeptic initiation.

**Meaning:**

The findings suggest that for women with severe mental illnesses and currently receiving stable treatment, continuing antidepressant treatment during pregnancy may be beneficial.

## Teaser

This cohort study investigates the association between antidepressant fill trajectories during pregnancy and postpartum psychiatric outcomes in Denmark and Norway.

## Introduction

Affective disorders are among the most common morbidities of pregnancy.^1,2,3^ Antidepressants constitute the mainstay of treatment for moderate to severe affective disorders for adults, including women of childbearing age.^4^ For individuals who respond to pharmaceutical treatment, long-term maintenance treatment with antidepressants is often needed to prevent recurrence.^5^

The treatment of pregnant women with antidepressants must balance possible risks of untreated mental disorders against fetal drug exposure.^4,6^ While psychiatric disorders before and during pregnancy are known to be associated with postpartum mental health,^7,8^ few studies have addressed how antidepressant use before and during pregnancy influences postpartum psychiatric outcomes. Some studies found that women who discontinued medications were more likely to experience a relapse than those who maintained their medications throughout the pregnancy,^9,10^ while others found no increased risk or reduced relapse risk.^11,12^ Additionally, a meta-analysis showed an increased relapse risk during pregnancy among antidepressant discontinuers compared with continuers (risk ratio, 1.74; 95% CI, 0.97-3.10).^13^ The risk was more pronounced among women with severe and recurrent depression (risk ratio, 2.30; 95% CI, 1.58-3.35). A recent study in Denmark found that pregnant women who discontinued antidepressants during but not before pregnancy had an increased risk of psychiatric emergency during pregnancy compared with continuers.^14^ Of note, antidepressant use patterns before and during pregnancy are complex, especially the timing of discontinuation.^15^ These studies usually used oversimplified exposure classification to assess the associated risks and are limited to 1 measurement of relapse.

In this study, we aimed to investigate the association between longitudinal trajectories of prescription fills for antidepressants during pregnancy and adverse psychiatric outcomes post partum in Denmark and Norway. We assessed 3 psychiatric outcomes from moderate to severe forms, including the initiation of psycholeptics, inpatient or emergency department visits for psychiatric disorders (abbreviated as psychiatric emergency), and self-harm, all observed within 1 year after delivery.

## Methods

This cohort study was conducted using data from population-based registers in Denmark and Norway,^16,17,18,19,20,21,22,23,24^ including prescription^19,25^ and medical birth registers^17,23^ (eTable 1 in Supplement 1). All live births and residents in the 2 countries are assigned a unique personal identification number, which can be used to link individual-level data between and within registers. The study was approved by the Regional Committee for Research Ethics in Southeastern Norway (2018/140/REK Sør–Øst) and the Data Protection Officer at the University of Oslo (58033) and by the Danish Data Protection Agency. By Norwegian and Danish law, no informed consent is required for register-based studies based on anonymized data. This study followed the Strengthening the Reporting of Observational Studies in Epidemiology (STROBE) reporting guideline.^26^

### Study Population

**Figure 1.**
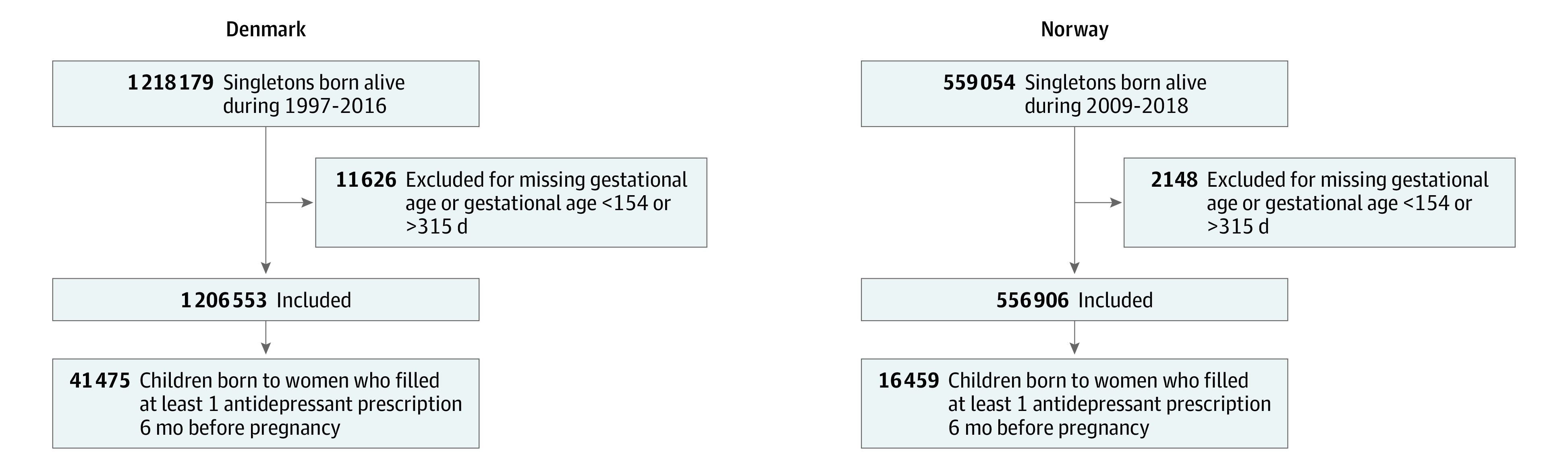
Flow Diagram Illustrating the Identification of the Study Population in Denmark and Norway

We identified all pregnancies ending in live-born singletons during 1997-2016 from the Danish Medical Birth Registry^17^ and 2009-2018 from the Norwegian Medical Birth Register.^23^ We restricted the sample to pregnancies that fulfilled the following criteria: (1) gestational age between 154 and 315 days and (2) at least 1 antidepressant prescription filled in the 6 months before pregnancy (Figure 1). Data on race and ethnicity are not collected in the source data used for this study. Altogether, 41 475 pregnancies in Denmark and 16 459 in Norway were eligible for the analyses. The analyses of initiation of psycholeptics were restricted to 38 554 pregnancies in Denmark and 11 250 pregnancies in Norway without a psycholeptic prescription filled in the 6 months before and during pregnancy.

### Antidepressant Treatment Before and During Pregnancy

We extracted information on filled antidepressant prescriptions from the Norwegian Prescription Database and the Danish National Prescription Register.^19,25^ The Anatomical Therapeutic Chemical (ATC) classification code N06A was used to identify antidepressants. Antidepressants were divided into the following classes: selective serotonin reuptake inhibitors (ATC code N06AB), serotonin-norepinephrine reuptake inhibitors (ATC code N06AX), and others (including any antidepressant other than selective serotonin or serotonin-norepinephrine reuptake inhibitors).

We estimated the expected duration of treatment for each prescription fill by using a simplified version of the prescription drug purchases to drug use period (PRE2DUP) method^15,27^ based on packaging parameters, leaflet information, and clinical guidelines for managing affective disorders requiring antidepressant treatment. The PRE2DUP method was used to calculate the end dates of each treatment course associated with each prescription based on the dispensation date and expected duration of treatment. The treatment courses were combined into antidepressant treatment periods if there was an overlap between more than 1 consecutive treatment. We defined antidepressant treatment status (yes or no) for 7-day intervals from 168 days (24 weeks) before pregnancy to 259 days (37 weeks) of gestation, totaling 61 weeks. For 4339 pregnancies (7.5%) with gestational age at delivery of less than 37 weeks, for each week from delivery to gestational week 37, treatment status was assigned the same value as the week before delivery.

### Potential Confounders

We identified the following potential confounders a priori using directed acyclic graphs^28^: age at pregnancy, primiparous, marital status, smoking during pregnancy, and Charlson Comorbidity Index (calculated based on 19 conditions; definition in the eMethods in Supplement 1). We used a history of self-harm (definition in the eMethods in Supplement 1) and psychiatric diagnoses at any time before pregnancy, including schizophrenia, bipolar disorder, depression, other mood disorders, and others (*International Classification of Diseases, Eighth Revision* [*ICD-8*] and *Tenth Revision* [*ICD-10*] codes listed in eTable 2 in Supplement 1); number of psychiatric emergencies; and coprescribed medications (opioid analgesics, antiseizure medications, antipsychotics, benzodiazepine/z-hypnotics, or anxiolytics; ATC codes listed in eTable 3 in Supplement 1) in the 6 months before pregnancy as a proxy for disease severity. In addition, we included filling prescriptions for 2 or more classes of antidepressants and having an average daily dose of an antidepressant greater than 1 fluoxetine dose equivalent (ie, 40 mg fluoxetine)^29^ in the 6 months before pregnancy as an additional proxy of the severity of mental illnesses.

### Outcomes of Interest

The outcomes of interest were initiating psycholeptics, psychiatric emergency, or self-harm within 1 year of delivery. Initiation of psycholeptics was defined as filling a psycholeptic (ATC code N05) prescription, including antipsychotics, anxiolytics, hypnotics, and sedatives, post partum without psycholeptic prescriptions filled in the 6 months before and during pregnancy. A psychiatric emergency was defined as having emergency department visits or inpatient treatment for mental disorders (*ICD-10* F codes).^14^ Self-harm was defined based on *ICD-8* and *ICD-10* codes derived from a validated coding algorithm^30^ (definition in the eMethods in Supplement 1). We considered psychiatric emergency and self-harm as the most severe psychiatric outcomes and initiation of psycholeptics as less severe than the other 2 outcomes.

### Statistical Analysis

#### Main Analyses

Between April 1 and October 30, 2022, the analyses were performed independently in Denmark and Norway according to a protocol established a priori. The relative risk estimates from the 2 countries were pooled using random-effects meta-analytic models.^31^ In each country, we applied the k-means for longitudinal data (KmL) trajectory modeling method to antidepressant treatment from 6 months before pregnancy until gestational week 37 to cluster the study population into groups.^15,32^ The KmL method has comparable performance to other methods (eg, group-based trajectory modeling) and is widely used to characterize longitudinal antidepressant use trajectories.^33,34^ We used the package kml in R and ran the k-means for 3 to 6 clusters 100 times each. The number of clusters was selected based on the maximization of 5 nonparametric quality criteria, the clinical relevance of the trajectories, and a minimum group size of 10%.^15,32^

We fitted Cox proportional hazards regression models to estimate unadjusted and weighted hazard ratios (HRs) of each outcome (initiation of psycholeptics, psychiatric emergency, and self-harm) associated with trajectory groups, using antidepressant continuers as the reference group. We used robust variance estimators to account for the dependence within pregnancies by the same woman. We used the time in days since delivery as the time scale and followed the women from birth until the first date of the outcome of interest or 365 days after delivery, whichever came first.

Approximately 8.0% of the values in Denmark and 14.0% in Norway were missing for potential confounders. We explored the patterns of missing data by exposure status, and under the assumption that the data were missing at random, we imputed missing data on covariates using multiple imputations with chained equations (10 replications).^35^ The imputation procedure included exposure variable, outcome variables, and covariates. Imputed data were used in all analyses. To account for potential confounders, we used propensity score–based methods with inverse probability of treatment weighting (IPTW).^36^ The propensity score was estimated by using a logistic regression model, where each discontinuing trajectory relative to continuers was the dependent variable and confounders were the independent variables. Data management and statistical analyses were performed using Stata/MP, version 16.0 (StataCorp LLC) and R, version 4.0.5 (R Foundation for Statistical Computing) software.

#### Sensitivity Analyses

We conducted 8 sensitivity analyses to test the robustness of the results. The first 5 were done in Norway and Denmark, while the last 3 were done only in Denmark. First and second, to further control for indications for treatment, we repeated our analyses among women with affective disorders and then again more stringently among women with major depression diagnosed any time before pregnancy (*ICD-8* and *ICD-10* codes in eTable 2 in Supplement 1). Third, some women had multiple eligible pregnancies and were included in the studies several times. To account for the dependency between pregnancies for the same woman, we restricted the analyses to first eligible pregnancies. Fourth, melatonin may be prescribed to treat insomnia, and its pharmacologic properties differ from those of other psychotropics. Therefore, we redefined initiation of psycholeptics as filling a psycholeptic prescription, excluding melatonin receptor agonists (ATC code N05 excluding N05CH). Fifth, to further account for the variation in gestational age, we restricted our analyses to 53 595 (92.5% of the study population) term deliveries. Sixth, to explore the potential residual confounding by socioeconomic status, we further adjusted for the highest education attained and income in the year of pregnancy. Seventh, to account for emigration and death occurring post partum, we repeated these analyses while censoring these events. Eighth, we further adjusted for imbalanced covariates after IPTW to see whether these would change the results.

## Results

A total of 57 934 pregnancies resulting in singleton births were included in the study, with 41 475 children born in Denmark (mean [SD] maternal age, 30.7 [5.3] years) and 16 459 in Norway (mean [SD] maternal age, 29.9 [5.5] years). The classes of antidepressant prescriptions filled before pregnancy are listed in eTable 4 in Supplement 1.

### Antidepressant Use Trajectories

Based on the maximization of 5 nonparametric quality criteria and clinical relevance, we selected the KmL models with 4 antidepressant fill trajectories in both countries as follows:

1. Early discontinuers: Women who decreased using antidepressants in the 6 months before pregnancy and discontinued proximal to the start of pregnancy.
2. Late discontinuers (previously stable users): Women who used antidepressants consistently throughout the 6 months before pregnancy and discontinued antidepressants in the second or third trimester.
3. Late discontinuers (short-term users): Women who increased using antidepressants in the 6 months before pregnancy and discontinued in the second or third trimester.
4. Continuers: Women who consistently used antidepressants during the 6 months before pregnancy and continued their antidepressant treatment throughout pregnancy.

**Figure 2.**
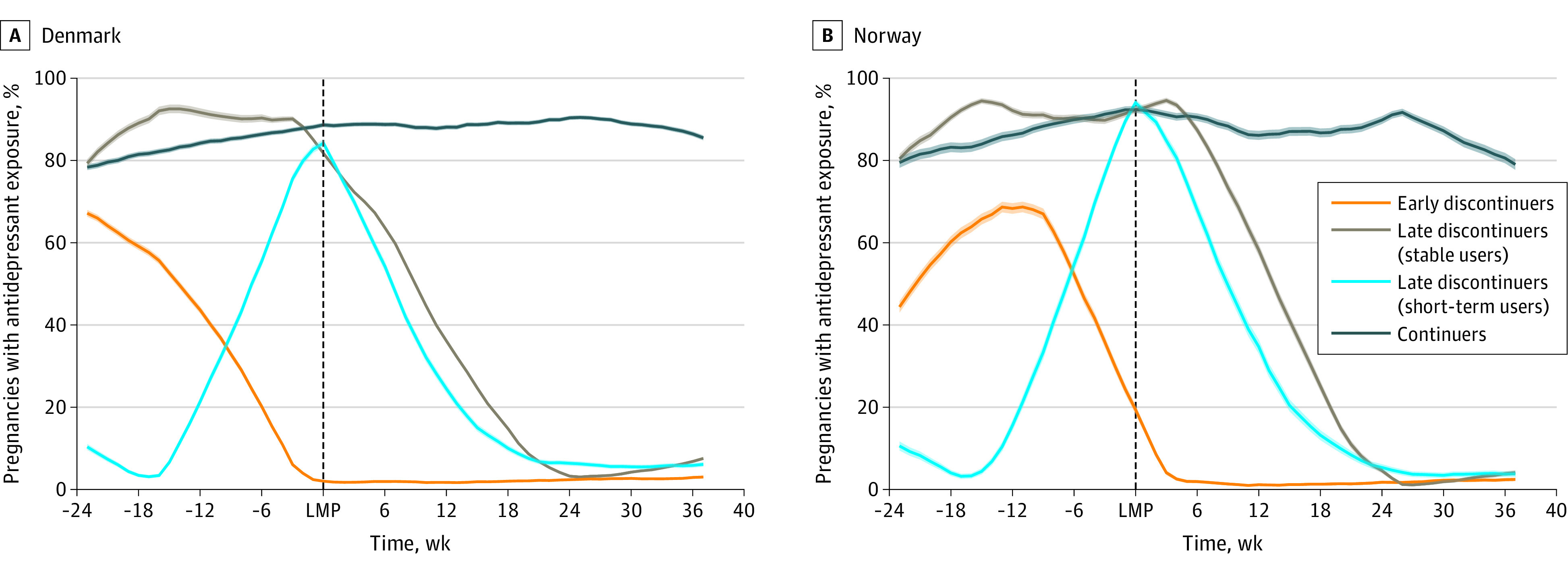
Antidepressant Fill Trajectories During Pregnancy in Denmark and Norway Early discontinuers decreased use of antidepressants in the 6 months before pregnancy and discontinued proximal to the start of pregnancy. Late discontinuers (previously stable users) used antidepressants consistently throughout the 6 months before pregnancy and discontinued in the second or third trimester. Late discontinuers (short-term users) increased use of antidepressants in the 6 months before pregnancy and discontinued in the second or third trimester. Continuers consistently used antidepressants during the 6 months before pregnancy and continued antidepressant treatment throughout pregnancy. The time window studied was 24 weeks before pregnancy and 37 weeks into pregnancy. Solid and dashed lines represent the trajectories and their 95% CIs. LMP indicates last menstrual period.

The 4 trajectory groups accounted for 31.3%, 21.5%, 15.9%, and 31.3%, respectively, of the study population in Denmark and 30.4%, 27.8%, 18.4%, and 23.4% in Norway (Figure 2).

Characteristics of women in the 4 trajectory groups are presented in the Table. Depression and neurotic, stress-related, and somatoform disorders were the most common psychiatric diagnoses before pregnancy for all 4 groups. Compared with the other trajectory groups, continuers were more likely to be older; to have a psychiatric diagnosis before pregnancy; to be on an average dose greater than 1 fluoxetine dose equivalent; and to fill prescriptions for antipsychotics, benzodiazepines, and antiseizure medications in the 6 months before pregnancy. After IPTW, the characteristics among these 4 trajectory groups were well balanced, except for the comparison between late discontinuers (short-term users) and continuers (eFigures 1-3 in Supplement 1).

**Table.** Maternal Characteristics by Trajectory Group in Denmark and Norway^a^

Characteristic	No. (%)^b^							
	Denmark				Norway			
	Early discontinuers (n = 12 983)	Late discontinuers (previously stable users) (n = 8920)	Late discontinuers (short-term users) (n = 6599)	Continuers (n = 12 973)	Early discontinuers (n = 5003)	Late discontinuers (previously stable users) (n = 4583)	Late discontinuers (short-term users) (n = 3027)	Continuers (n = 3846)
**Demographic factors**									
Age at conception, y								
≤24	2352 (18.1)	1427 (16.0)	1415 (21.4)	1324 (10.2)	985 (19.7)	751 (16.4)	677 (22.4)	454 (11.8)
25-29	4046 (31.2)	2666 (29.9)	1950 (29.5)	3636 (28.0)	1549 (31.0)	1487 (32.5)	919 (30.4)	1094 (28.5)
30-34	4134 (31.8)	2869 (32.2)	1925 (29.2)	4693 (36.2)	1485 (29.7)	1371 (29.9)	851 (28.1)	1274 (33.1)
≥35	2451 (18.9)	1958 (22.0)	1309 (19.8)	3320 (25.6)	984 (19.7)	974 (21.2)	580 (19.2)	1024 (26.6)
Primiparous	5862 (45.2)	4265 (47.8)	2768 (41.9)	5945 (45.8)	2274 (45.4)	2255 (49.2)	1274 (42.1)	1630 (42.4)
Smoking during pregnancy								
Yes	3610 (27.8)	2460 (27.6)	2173 (32.9)	3313 (25.5)	840 (16.8)	795 (17.4)	568 (18.8)	636 (16.5)
No	8626 (66.4)	6033 (67.6)	4077 (61.8)	9257 (71.4)	3460 (69.2)	3155 (68.8)	2015 (66.6)	2724 (70.8)
Missing	747 (5.8)	427 (4.8)	349 (5.3)	403 (3.1)	703 (14.0)	633 (13.8)	444 (14.7)	486 (12.6)
Marital status in the year of delivery								
Married or cohabiting	9719 (74.9)	6525 (73.2)	4636 (70.3)	10 070 (77.6)	4338 (86.7)	3925 (85.6)	2572 (85.0)	3411 (88.7)
Single, divorced, or widowed	2883 (22.2)	2116 (23.7)	1748 (26.5)	2368 (18.3)	630 (12.6)	593 (12.9)	417 (13.8)	395 (10.3)
Missing	381 (2.9)	279 (3.1)	215 (3.3)	535 (4.1)	35 (0.7)	65 (1.4)	38 (1.3)	40 (1.0)
Highest education in the year of pregnancy								
Mandatory school	4530 (34.9)	2824 (31.7)	2740 (41.5)	3298 (25.4)	NA	NA	NA	NA
Above mandatory school	8056 (62.1)	5880 (65.9)	3614 (54.8)	9398 (72.4)	NA	NA	NA	NA
Missing	397 (3.1)	216 (2.4)	245 (3.7)	277 (2.1)	NA	NA	NA	NA
Age-specific income in the year of pregnancy, quartile								
Lowest	2507 (19.3)	1786 (20.0)	1406 (21.3)	2723 (21.0)	NA	NA	NA	NA
Second	3523 (27.1)	2424 (27.2)	1759 (26.7)	3263 (25.2)	NA	NA	NA	NA
Third	2851 (22.0)	1877 (21.0)	1381 (20.9)	2677 (20.6)	NA	NA	NA	NA
Highest	2649 (20.4)	1640 (18.4)	1298 (19.7)	2166 (16.7)	NA	NA	NA	NA
Missing	1453 (11.2)	1193 (13.4)	755 (11.4)	2144 (16.5)	NA	NA	NA	NA
Calendar year of pregnancy								
1996-2005	4596 (35.4)	2738 (30.7)	2319 (35.1)	2692 (20.8)	0	0	0	0
2006-2010	4212 (32.4)	2807 (31.5)	2183 (33.1)	5484 (42.3)	1035 (20.7)	863 (18.8)	634 (21.0)	646 (16.8)
2011-2018	4175 (32.2)	3375 (37.8)	2097 (31.8)	4797 (37.0)	3968 (79.3)	3720 (81.2)	2393 (79.0)	3200 (83.2)
**Clinical factors**									
Psychiatric diagnosis any time before pregnancy								
Substance abuse disorder	344 (2.6)	310 (3.5)	206 (3.1)	503 (3.9)	223 (4.5)	206 (4.5)	141 (4.7)	198 (5.2)
Schizophrenia	308 (2.4)	261 (2.9)	170 (2.6)	494 (3.8)	45 (0.9)	59 (1.3)	28 (0.9)	42 (1.1)
Bipolar disorder	103 (0.8)	106 (1.2)	42 (0.6)	281 (2.2)	131 (2.6)	200 (4.4)	83 (2.7)	258 (6.7)
Depression	1973 (15.2)	1852 (20.8)	945 (14.3)	3847 (29.7)	1261 (25.2)	1493 (32.6)	711 (23.5)	1345 (35.0)
Other mood disorder	139 (1.1)	149 (1.7)	59 (0.9)	293 (2.3)	107 (2.1)	132 (2.9)	51 (1.7)	119 (3.1)
Neurotic, stress-related, and somatoform disorders	2598 (20.0)	2276 (25.5)	1353 (20.5)	4282 (33.0)	1619 (32.4)	1910 (41.7)	975 (32.2)	1868 (48.6)
Personality disorders	1345 (10.4)	1228 (13.8)	675 (10.2)	2313 (17.8)	258 (5.2)	334 (7.3)	137 (4.5)	359 (9.3)
Child-onset disorders	298 (2.3)	265 (3.0)	175 (2.7)	355 (2.7)	263 (5.3)	266 (5.8)	192 (6.3)	224 (5.8)
Other mental illnesses	771 (5.9)	677 (7.6)	340 (5.2)	1249 (9.6)	573 (11.5)	718 (15.7)	381 (12.6)	628 (16.3)
Self-harm before pregnancy	969 (7.5)	790 (8.9)	578 (8.8)	1180 (9.1)	0	0	0	0
No. of psychiatric emergencies within 6 mo before pregnancy								
0	12 704 (97.9)	8643 (96.9)	6309 (95.6)	12 549 (96.7)	4820 (96.3)	4347 (94.8)	2913 (96.2)	3677 (95.6)
1	104 (0.8)	98 (1.1)	122 (1.8)	172 (1.3)	67 (1.3)	83 (1.8)	39 (1.3)	51 (1.3)
2	92 (0.7)	75 (0.8)	95 (1.4)	121 (0.9)	16 (0.3)	36 (0.8)	15 (0.5)	18 (0.5)
≥3	83 (0.6)	104 (1.2)	73 (1.1)	131 (1.0)	100 (2.0)	117 (2.6)	100 (2.6)	60 (2.0)
No. of classes of antidepressant treatment within 6 mo before pregnancy								
1	12 369 (95.3)	7896 (88.5)	6211 (94.1)	11 439 (88.2)	4797 (95.9)	4205 (91.8)	2861 (94.5)	3547 (92.2)
2 and 3	614 (4.7)	1024 (11.5)	388 (5.9)	1534 (11.8)	206 (4.1)	378 (8.2)	166 (5.5)	299 (7.8)
Average daily dose of antidepressants in 6 mo before pregnancy, FDE^c^								
<1	12 947 (99.7)	8071 (90.5)	NA	10 469 (80.7)	4865 (97.2)	3924 (85.6)	NA	2993 (77.8)
≥1	36 (0.3)	849 (9.5)	NA	2504 (19.3)	138 (2.8)	659 (14.4)	NA	853 (22.2)
Coprescribed medications within 6 mo before pregnancy								
Antipsychotics	565 (4.4)	699 (7.8)	358 (5.4)	1169 (9.0)	337 (6.7)	402 (8.8)	215 (7.1)	365 (9.5)
Opioids	879 (6.8)	631 (7.1)	461 (7.0)	811 (6.3)	732 (14.6)	600 (13.1)	510 (16.9)	516 (13.4)
Anxiolytics	38 (0.3)	34 (0.4)	20 (0.3)	52 (0.4)	103 (2.1)	113 (2.5)	77 (2.5)	97 (2.5)
Benzodiazepines	1316 (10.1)	1091 (12.2)	934 (14.2)	1674 (12.9)	876 (17.5)	839 (18.3)	652 (21.5)	800 (20.8)
Antiseizure medications	370 (2.8)	442 (5.0)	187 (2.8)	792 (6.1)	263 (5.3)	313 (6.8)	145 (4.8)	316 (8.2)
Charlson Comorbidity Index before pregnancy								
0	11 414 (87.9)	7811 (87.6)	5793 (87.8)	11 237 (86.6)	4779 (95.5)	4370 (95.4)	2905 (96.0)	3641 (94.7)
1	1186 (9.1)	849 (9.5)	614 (9.3)	1301 (10.0)	202 (4.0)	182 (4.0)	109 (3.6)	183 (4.8)
≥2	383 (3.0)	260 (2.9)	192 (2.9)	435 (3.4)	22 (0.5)	31 (0.7)	13 (0.4)	22 (0.6)

Abbreviations: FDE, fluoxetine dose equivalent; NA, not available.

^a^ Early discontinuers decreased use of antidepressants in the 6 months before pregnancy and discontinued proximal to the start of pregnancy. Late discontinuers (previously stable users) used antidepressants consistently throughout the 6 months before pregnancy and discontinued antidepressants in the second or third trimester. Late discontinuers (short-term users) increased use of antidepressants in the 6 months before pregnancy and discontinued in the second or third trimester. Continuers consistently used antidepressants during the 6 months before pregnancy and continued antidepressant treatment throughout pregnancy.

^b^ Numbers in parentheses represent the proportion of each category among the total number of pregnancies in a cluster.

^c^ Fewer than 5 women had a daily dose of antidepressants >1 FDE among late discontinuers (short-term users).

### Association of Antidepressant Use Trajectories With Adverse Psychiatric Outcomes

#### Initiation of Psycholeptics

**Figure 3.**
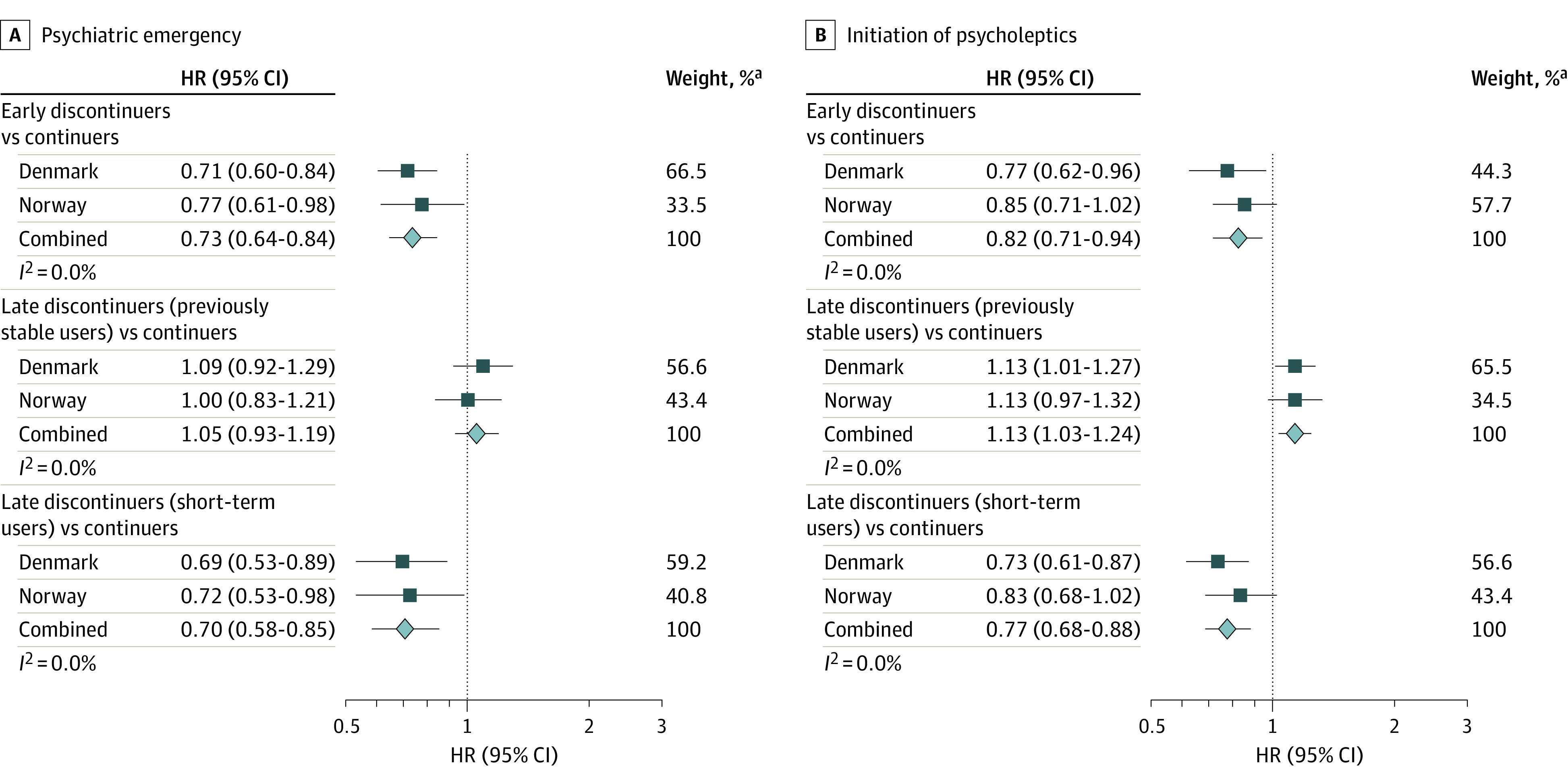
Antidepressant Treatment Trajectories and Hazard Ratios (HRs) of Postpartum Psychiatric Emergencies and Initiation of Psycholeptics Early discontinuers decreased use of antidepressants in the 6 months before pregnancy and discontinued proximal to the start of pregnancy. Late discontinuers (previously stable users) used antidepressants consistently throughout the 6 months before pregnancy and discontinued in the second or third trimester. Late discontinuers (short-term users) increased use of antidepressants in the 6 months before pregnancy and discontinued in the second or third trimester. Continuers consistently used antidepressants during the 6 months before pregnancy and continued antidepressant treatment throughout pregnancy. ^a^Weights are from random-effects analysis.

Of 49 804 women with no psycholeptic medication from 6 months before pregnancy until delivery, 2227 of 38 554 (5.8%) in the Danish cohort and 1128 of 11 250 (10.0%) in the Norwegian cohort initiated psycholeptics in the postpartum period (psycholeptic drug classes listed in eTable 5 in Supplement 1). Early discontinuers and late discontinuers (short-term users) had a lower probability of initiating psycholeptics compared with continuers, while late discontinuers (previously stable users) had a 13% increased probability of postpartum initiation of psycholeptics (pooled HR, 1.13; 95% CI, 1.03-1.24) (Figure 3).

#### Psychiatric Emergency

Altogether, 2.6% (1078 of 41 475) and 4.3% (705 of 16 459) of pregnant women in Denmark and Norway, respectively, experienced a postpartum psychiatric emergency. Early discontinuers and late discontinuers (short-term users) had a lower risk of postpartum psychiatric emergencies compared with continuers, with a pooled HR of 0.73 (95% CI, 0.64-0.84) and 0.70 (95% CI, 0.58-0.85), respectively, while the risk did not differ between late discontinuers (previously stable users) and continuers (pooled HR, 1.05; 95% CI, 0.93-1.19) (Figure 3). The magnitude of the associations was comparable between the 2 countries (eTable 6 in Supplement 1).

#### Self-Harm

Approximately 0.3% (126 of 41 475) of pregnant women in the Danish cohort had recorded self-harm after delivery. The risk of self-harm did not differ between the other trajectory groups and continuers (early discontinuers vs continuers: HR, 0.69 [95% CI, 0.32-1.14]; late discontinuers [previously stable users] vs continuers: HR, 1.04 [95% CI, 0.65-1.68]; and late discontinuers [short-term users] vs continuers: HR, 0.54 [95% CI, 0.28-1.06]).

### Sensitivity Analyses

**Figure 4.**
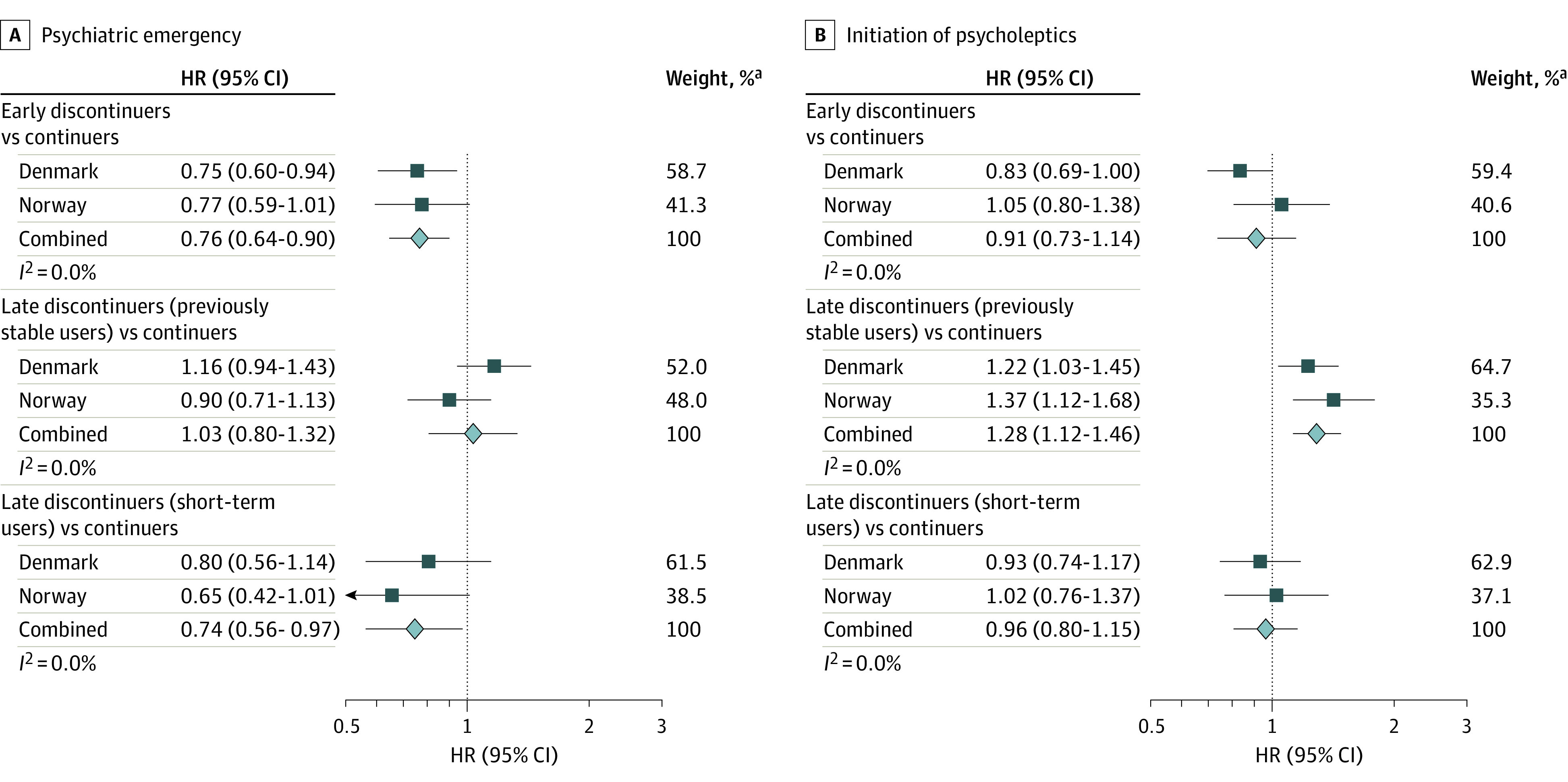
Antidepressant Treatment Trajectories and Hazard Ratios (HRs) of Postpartum Psychiatric Emergencies and Initiation of Psycholeptics Among Women With Preexisting Affective Disorders Early discontinuers decreased use of antidepressants in the 6 months before pregnancy and discontinued proximal to the start of pregnancy. Late discontinuers (previously stable users) used antidepressants consistently throughout the 6 months before pregnancy and discontinued antidepressants in the second or third trimester. Late discontinuers (short-term users) increased use of antidepressants in the 6 months before pregnancy and discontinued in the second or third trimester. Continuers consistently used antidepressants during the 6 months before pregnancy and continued antidepressant treatment throughout pregnancy. ^a^Weights are from random-effects analysis.

In the analyses restricted to women with previous affective disorders and those with major depressive disorders, the results were comparable with those in the primary analyses, although late discontinuers (previously stable users) had a slightly higher risk of initiation of psycholeptics than continuers (pooled HRs, 1.28 [95% CI, 1.12-1.46] and 1.31 [95% CI, 1.09-1.58], respectively) than in the primary analysis (pooled HR, 1.13; 95% CI, 1.03-1.24) (Figure 4; eTables 7 and 8 in Supplement 1). The results remained similar when we repeated our analyses in the first eligible pregnancies or term pregnancies (eTables 9 and 10 in Supplement 1) and when redefining initiation of psycholeptics excluding melatonin (eTable 11 in Supplement 1), further accounting for censoring due to emigration and death, additionally adjusting for education and income status, and further controlling for imbalanced covariates after IPTW (eTable 12 in Supplement 1).

## Discussion

In this cohort study using registry data comprising approximately 60 000 pregnancies, we identified 4 similar antidepressant prescription fill trajectories during pregnancy in Denmark and Norway: early discontinuers, late discontinuers (previously stable users), late discontinuers (short-term users), and continuers. Findings from early discontinuers and late discontinuers (short-term users) were associated with a lower risk of psychiatric emergencies and a lower probability of postpartum psycholeptic initiation compared with continuers. A moderately elevated probability of postpartum psycholeptic initiation was found in late discontinuers (previously stable users) compared with continuers. There was no evidence that antidepressant discontinuation was associated with increased risks of the most severe adverse psychiatric outcomes post partum.

We found that the timing of antidepressant discontinuation may play an important role in postpartum psychiatric outcomes, with early discontinuation and late discontinuation (short-term use) associated with a lower risk of all 3 psychiatric outcomes and late discontinuation (previously stable use) with a moderately higher probability of initiation of psycholeptics compared with continuation. Our findings are in line with a prior study in Denmark that reported a reduced risk of psychiatric emergencies among women who discontinued antidepressant use early in pregnancy and a higher risk associated with discontinuation during pregnancy compared with continuers (HR 1.25; 95% CI, 1.00-1.55), while the postpartum risk was not significant.^14^ Women who discontinued antidepressants early in pregnancy or discontinued late in pregnancy after short-term use may have less severe underlying disorders and can successfully stop their medications. On the other hand, those who discontinued late in pregnancy after long-term use may have had more severe episodes and may benefit from individual assessment before discontinuation. Although there is no available information on the reasons for discontinuing, we speculate that women might discontinue antidepressants in late pregnancy due to fear of neonatal adaptation syndrome or neonatal persistent pulmonary hypertension.^37,38^ Abrupt antidepressant discontinuation may lead to withdrawal symptoms that can directly lead to a renewed need for medication treatment. Psycholeptics are often used as an add-on therapy to improve treatment response or to treat withdrawal symptoms.^39^ Initiation of psycholeptics can be a proxy for potential moderate disease relapse or exacerbation or inadequate treatment response to antidepressant treatment.^39,40^

Characterizing individuals with increased psychiatric risks post partum is crucial for treatment counseling. For instance, both late discontinuers (previously stable users) and continuers received a higher antidepressant dose before pregnancy than early discontinuers and late discontinuers (short-term users). Dosage information may be easily extracted from the medical records, especially when the patient has been followed up by the same clinician during the consultation. We did not have detailed data on the reasons for discontinuing the antidepressant treatment. Some women may have received nonpharmacologic treatment (eg, psychotherapy). Our findings provide no evidence that current guidelines for antidepressant treatment during pregnancy should be changed.

### Strengths and Limitations

Our study is based on a large representative population by combining data from 2 countries. Because information on filled antidepressant prescriptions and data on psychiatric emergency, self-harm, and psycholeptic initiation were collected as part of the health care system, information bias was minimized. We modeled longitudinal antidepressant exposure using the KmL method, which overcomes the limitation of using a fixed time window and revealed different discontinuation patterns.^33^ We minimized the confounding by indication by restricting the analysis to pregnant women with affective disorders and pregnant women with major depression before pregnancy and included covariates to address maternal severity of psychiatric disorders.

Our study has some limitations. First, the analysis of self-harm risk was based on Danish data only, and we could not draw an accurate conclusion based on the small sample size. Second, although we included several variables as a proxy of disease severity, eg, previous psychiatric diagnoses and coprescribed medications, residual confounding by treatment indications may still prevail. However, the sensitivity analyses among pregnant women with preexisting affective disorders and women with major depression are generally in line with the main findings. Third, we do not know why pregnant women in our cohorts discontinued their treatment. Fourth, although we used the PRE2DUP method to estimate the duration of treatment, these estimations may still deviate from the actual duration of treatment, resulting in potential misclassification. However, the method has high validity, and the misclassification is minimal.^15,27^

## Conclusions

In this population-based cohort study in Denmark and Norway, we found a moderately elevated probability of postpartum initiation of psycholeptics in women who were late discontinuers (previously stable users) during pregnancy but a lower probability of psychiatric emergencies and psycholeptic initiation in early discontinuers and late discontinuers (short-term users) compared with continuers. These findings suggest that individuals with severe mental illnesses who are currently on stable treatment may benefit from personalized treatment counseling.

## Notes

Supplement 1.
eMethods.
**eTable 1.** Data Sources Used in the Study **eTable 2.** *International Classification of Diseases, Eighth Revision* (*ICD-8*) and *Tenth Revision* (*ICD-10*) Codes for Psychiatric Diagnoses Before Pregnancy **eTable 3.** Anatomical Therapeutic Council (ATC) Classification Codes for Comedications **eTable 4.** Classes of Antidepressant Treatment Before Pregnancy (93 005 Prescriptions in 41 475 Pregnancies in Denmark and 30 546 Prescriptions in 16 459 Pregnancies in Norway) **eTable 5.** Classes of Psycholeptic Medications Used Postpartum in Denmark (n = 2227) and Norway (n = 1128) **eTable 6.** Associations Between Antidepressant Treatment During Pregnancy and Maternal Mental Health Outcomes in the Postpartum Year in 41 475 Pregnancies in Denmark and 16 459 Pregnancies in Norway **eTable 7.** Associations Between Antidepressant Treatment During Pregnancy and Maternal Mental Health Outcomes in the Postpartum Year in Norway: Analysis Limited to the Population Having a Diagnosis of Affective Disorder in the 1 Year Before Pregnancy (14 726 in Denmark and 8542 in Norway) **eTable 8.** Associations Between Antidepressant Treatment During Pregnancy and Maternal Mental Health Outcomes in the Postpartum Year in Norway: Analysis Limited to the Population With a Diagnosis of Major Depression in the 1 Year Before Pregnancy (8617 in Denmark and 4810 in Norway) **eTable 9.** Associations Between Antidepressant Treatment During Pregnancy and Maternal Mental Health Outcomes in the Postpartum Year in 35 463 Eligible Pregnancies in Denmark and 15 019 Eligible Pregnancies in Norway **eTable 10.** Associations Between Antidepressant Treatment During Pregnancy and Maternal Mental Health Outcomes in the Postpartum Year in Denmark Among Term Pregnancies (38 267 in Denmark and 15 328 in Norway) **eTable 11.** Associations Between Antidepressant Treatment During Pregnancy and Initiation of Psycholeptic Medications, Excluding Melatonin, in the Postpartum Year in 38 587 Pregnancies in Denmark and 11 588 Pregnancies in Norway With No Psycholeptic Use From 6 Months Before Pregnancy Until Delivery **eTable 12.** Associations Between Antidepressant Treatment During Pregnancy and Maternal Mental Health Outcomes in the Postpartum Year in Denmark **eFigure 1.** Standardized Mean Difference in Confounding Factors Before and After Inverse Probability of Treatment Weighting (IPTW) in Early Discontinuers vs Continuers **eFigure 2.** Standardized Mean Difference in Confounding Factors Before and After Inverse Probability of Treatment Weighting (IPTW) in Late Discontinuers (Previously Stable Users) vs Continuers **eFigure 3.** Standardized Mean Difference in Confounding Factors Before and After Inverse Probability of Treatment Weighting (IPTW) in Late Discontinuers (Short-Term Users) vs Continuers
eReferences

Supplement 2.
Data Sharing Statement
