# Supplementary material for: Timing of Antidepressant Discontinuation During Pregnancy and Postpartum Psychiatric Outcomes in Denmark and Norway
Source: JAMA Psychiatry. 2023 Mar 8;80(5):441–50. doi: 10.1001/jamapsychiatry.2023.0041 (PMC9996461; doi:10.1001/jamapsychiatry.2023.0041)
Supplement: Supplement 1. — eMethods. eTable 1. Data Sources Used in the Study eTable 2. International Classification of Diseases, Eighth Revision (ICD-8) and Tenth Revision (ICD-10) Codes for Psychiatric Diagnoses Before Pregnancy eTable 3. Anatomical Therapeutic Council (ATC) Classification Codes for Comedications eTable 4. Classes of Antidepressant Treatment Before Pregnancy (93 005 Prescriptions in 41 475 Pregnancies in Denmark and 30 546 Prescriptions in 16 459 Pregnancies in Norway) eTable 5. Classes of Psycholeptic Medications Used Postpartum in Denmark (n = 2227) and Norway (n = 1128) eTable 6. Associations Between Antidepressant Treatment During Pregnancy and Maternal Mental Health Outcomes in the Postpartum Year in 41 475 Pregnancies in Denmark and 16 459 Pregnancies in Norway eTable 7. Associations Between Antidepressant Treatment During Pregnancy and Maternal Mental Health Outcomes in the Postpartum Year in Norway: Analysis Limited to the Population Having a Diagnosis of Affective Disorder in the 1 Year Before Pregnancy (14 726 in Denmark and 8542 in Norway) eTable 8. Associations Between Antidepressant Treatment During Pregnancy and Maternal Mental Health Outcomes in the Postpartum Year in Norway: Analysis Limited to the Population With a Diagnosis of Major Depression in the 1 Year Before Pregnancy (8617 in Denmark and 4810 in Norway) eTable 9. Associations Between Antidepressant Treatment During Pregnancy and Maternal Mental Health Outcomes in the Postpartum Year in 35 463 Eligible Pregnancies in Denmark and 15 019 Eligible Pregnancies in Norway eTable 10. Associations Between Antidepressant Treatment During Pregnancy and Maternal Mental Health Outcomes in the Postpartum Year in Denmark Among Term Pregnancies (38 267 in Denmark and 15 328 in Norway) eTable 11. Associations Between Antidepressant Treatment During Pregnancy and Initiation of Psycholeptic Medications, Excluding Melatonin, in the Postpartum Year in 38 587 Pregnancies in Denmark and 11 588 Pregnancies in Norway With N [file jamapsychiatry-e230041-s001.pdf]

Supplemental Online Content

Trinh NTH, Munk-Olsen T, Wray NR, et al. Timing of antidepressant discontinuation during pregnancy and psychiatric outcomes postpartum. *JAMA Psychiatry*. Published online March 8, 2023. doi:10.1001/jamapsychiatry.2023.0041

eMethods.

eTable 1. Data Sources Used in the Study

eTable 2. *International Classification of Diseases, Eighth Revision* (*ICD-8*) and *Tenth Revision* (*ICD-10*) Codes for Psychiatric Diagnoses Before Pregnancy

eTable 3. Anatomical Therapeutic Council (ATC) Classification Codes for Comedications

eTable 4. Classes of Antidepressant Treatment Before Pregnancy (93 005 Prescriptions in 41 475 Pregnancies in Denmark and 30 546 Prescriptions in 16 459 Pregnancies in Norway)

eTable 5. Classes of Psycholeptic Medications Used Postpartum in Denmark (n = 2227) and Norway (n =1128)

eTable 6. Associations Between Antidepressant Treatment During Pregnancy and Maternal Mental Health Outcomes in the Postpartum Year in 41 475 Pregnancies in Denmark and 16 459 Pregnancies in Norway

eTable 7. Associations Between Antidepressant Treatment During Pregnancy and Maternal Mental Health Outcomes in the Postpartum Year in Norway: Analysis Limited to the Population Having a Diagnosis of Affective Disorder in the 1 Year Before Pregnancy (14 726 in Denmark and 8542 in Norway)

eTable 8. Associations Between Antidepressant Treatment During Pregnancy and Maternal Mental Health Outcomes in the Postpartum Year in Norway: Analysis Limited to the Population With a Diagnosis of Major Depression in the 1 Year Before Pregnancy (8617 in Denmark and 4810 in Norway)

eTable 9. Associations Between Antidepressant Treatment During Pregnancy and Maternal Mental Health Outcomes in the Postpartum Year in 35 463 Eligible Pregnancies in Denmark and 15 019 Eligible Pregnancies in Norway

eTable 10. Associations Between Antidepressant Treatment During Pregnancy and Maternal Mental Health Outcomes in the Postpartum Year in Denmark Among Term Pregnancies (38 267 in Denmark and 15 328 in Norway)

eTable 11. Associations Between Antidepressant Treatment During Pregnancy and Initiation of Psycholeptic Medications, Excluding Melatonin, in the Postpartum Year in 38 587 Pregnancies in Denmark and 11 588 Pregnancies in Norway With No Psycholeptic Use From 6 Months Before Pregnancy Until Delivery

eTable 12. Associations Between Antidepressant Treatment During Pregnancy and Maternal Mental Health Outcomes in the Postpartum Year in Denmark

eFigure 1. Standardized Mean Difference in Confounding Factors Before and After Inverse Probability of Treatment Weighting (IPTW) in Early Discontinuers vs Continuers

eFigure 2. Standardized Mean Difference in Confounding Factors Before and After Inverse Probability of Treatment Weighting (IPTW) in Late Discontinuers (Previously Stable Users) vs Continuers

eFigure 3. Standardized Mean Difference in Confounding Factors Before and After Inverse Probability of Treatment Weighting (IPTW) in Late Discontinuers (Short-Term Users) vs Continuers

eReferences

This supplemental material has been provided by the authors to give readers additional information about their work.

© 2023 American Medical Association. All rights reserved.

eMethods

Ascertainment of the start of pregnancy

we determined the start of pregnancy by subtracting gestational length in days from the date of delivery. The gestational length was mainly ascertained through the first- or second-trimester ultrasound scan.[1,2] If ultrasound data were unavailable, the first day of the last menstrual period (LMP) reported by the woman was used to indicate the start of pregnancy.[3]

Definition of self-harm

Self-harm before the time of delivery was defined as inpatient or outpatient contact for self-harm identified from the Danish Psychiatric Central Research Register and the Danish National Patient Register.[4] Individuals were considered to have made a suicide attempt if they fulfilled one of the following criteria:
(1) The main diagnosis of suicide attempt or deliberate self-harm (ICD-8 code: E950–E959; ICD-10: X60–84)
(2) The main diagnosis of poisoning with weak analgesics, epileptic drugs, or carbon monoxide (ICD-10: T39, T42, T43, and T58)
(3) Hospital contacts where the primary diagnosis is a psychiatric disorder (any from the ICD-10 F chapter and 290–315 in ICD-8) and the secondary diagnosis is intoxication (ICD-10: T36–T50 and T52–T60) or lesions at the forearm, wrist, or hand (ICD-10: S51, S55, S59, S61, S65, and S69)

Charlson comorbidity index[5]

(1) Comorbidity category assigned a score of 1: Myocardial infarction, congestive heart failure, peripheral vascular disease, cerebrovascular disease, dementia, chronic pulmonary disease, connective tissue disease, ulcer disease, mild liver disease, type 1 and type 2 diabetes
(2) Comorbidity category assigned a score of 2: Hemiplegia, moderate to severe renal disease, diabetes with end-organ damage type 1 and type 2, any tumor, leukemia, and lymphoma
(3) Comorbidity category assigned a score of 3: Moderate to severe liver disease
(4) Comorbidity category assigned a score of 6: Metastatic solid tumor and AIDs

Data sharing statement

The study was based on the nationwide registers in Denmark and Norway. According to Danish and Norwegian legislation, individual-level data can be accessed only through secure servers, Denmark Statistics and the Service for Sensitive Data – TSD at the University of Oslo in Norway, where download or export of individual-level information is prohibited. In Norway, data are available for the researchers upon request to the registry holders, provided legal and ethical approvals. In Denmark, only aggregated data can be shared to ensure complete anonymity and protection of individuals included in the studies.

© 2023 American Medical Association. All rights reserved.

eTable 1. Data Sources Used in the Study

Data source	Obtained information	Years
**Denmark**		
The Danish Civil Registration System	Emigration and death	1968–2019
The Danish Medical Birth Register	Study population and birth outcomes	1973–2016
The Danish National Prescription Registry	Medication use	1995–2019
The Danish Psychiatric Central Research Register	Maternal underlying conditions and outcomes	1969– 2019, *outpatients* are included since 1995
The Danish National Patient Register	Maternal underlying conditions and outcomes	1977–2019, *outpatients* are included since 1995
**Norway**		
Medical Birth Registry of Norway	Study population and birth outcomes	1967–2019
Norwegian Prescription Database	Medication use	2004–2019
Norwegian control and payment of health reimbursements	Secondary care (i.e., consultations with specialists)	2006–2019
The Norwegian National Patient Registry	Secondary care and hospitalization	2008–2019

© 2023 American Medical Association. All rights reserved.

eTable 2. *International Classification of Diseases, Eighth Revision* (*ICD-8*) and *Tenth Revision* (*ICD-10*) Codes for Psychiatric Diagnoses Before Pregnancy

**Name of disorders**	**ICD-8 codes**	**ICD-10 codes**
Any psychiatric disorders	290–309	F00–F99 excluding F70–F79
Substance abuse disorder	291.X9, 294.39, 303.X9, 303.20, 303.28, 303.90, and 304.X9	F10–F19
Schizophrenia and related disorders, abbreviated hereafter as schizophrenia	295.X9, 296.89, 297.X9, 298.29–298.99, 299.04, 299.05, 299.09, and 301.83	F20–F29
Bipolar disorder	296.19, 296.39, and 298.19	F30–31
Depression	296.09, 296.29, 298.09, and 300.49	F32–33
Other mood disorders except for bipolar and unipolar disorders;	296.X9 and 301.19 excluding 296.09, 296.19, 296.29, and 296.39	F34–39
Neurotic, stress-related, and somatoform disorders	300.X9, 305.X9, 305.68, and 307.99 excluding 300.49	F40–F48
Personality disorders	300.19, 301.49, 301.59, 301.69, 301.79, 301.80, 301.81, 301.82, 301.83, and 301.84	F60–F69
Behavioral and emotional disorders with onset usually occurring in childhood and adolescence	306.X9, and 308.0X	F90–F98

Affective disorders are defined as having a diagnosis of bipolar disorder, depression, other mood disorders, or neurotic, stress-related, and somatoform disorders. The following ICD-codes were used: ICD-8 codes 296.x9, 298.09, 298.19, 300.49, 301.19, 300.x9, 305.x9, 305.68, 307.99 excluding 296.89; ICD-10 codes F30–F39, F40–F48.

© 2023 American Medical Association. All rights reserved.

eTable 3. Anatomical Therapeutic Council (ATC) Classification Codes for Comedications

**Group of comedications**	**ATC codes**
Opioid analgesics	N02A
Antiseizure medications	N03A
Antipsychotics	N05A
Benzodiazepine/z-hypnotics	N05BA, N05CD, N05CF
Anxiolytics	N05B except for N05BA

© 2023 American Medical Association. All rights reserved.

eTable 4. Classes of Antidepressant Treatment Before Pregnancy (93 005 Prescriptions in 41 475 Pregnancies in Denmark and 30 546 Prescriptions in 16 459 Pregnancies in Norway)

Antidepressant	ATC code	Denmark		Norway	
		No. of pregnancies	%	No. of pregnancies	%
**Selective serotonin reuptake inhibitors (SSRIs)**	**N06AB**	**32,329**	**77.9**	**10,270**	**62.4**
Citalopram	N06AB04	28,055	67.6	927	5.6
Sertraline	N06AB06	16,049	38.7	1,829	11.1
Fluoxetine	N06AB03	9,327	22.5	1,054	6.4
Escitalopram	N06AB10	7,367	17.8	6,501	39.5
Paroxetine	N06AB05	6,424	15.5	345	2.1
Fluvoxamine	N06AB08	85	0.2	17	0.1
More than one SSRI	-	0	0.0	9	0.1
**Serotonin-norepinephrine reuptake inhibitors (SNRIs)**	**N06AX**	**9,878**	**23.8**	**3,701**	**22.5**
Venlafaxine	N06AX16	10,900	26.3	1,567	9.5
Mirtazapine	N06AX11	4,221	10.2	938	5.7
Duloxetine	N06AX21	2,923	7.0	127	0.8
Mianserin	N06AX03	937	2.3	688	4.2
Bupropion	N06AX12	562	1.4	517	3.1
Agomelatine	N06AX22	412	1.0	0	0.0
Reboxetine	N06AX18	154	0.4	21	0.1
Nefazodone	N06AX06	66	0.2	0	0.0
Vortioxetine	N06AX26	28	0.1	73	0.4
Oxitriptan	N06AX01	0	0	11	0.1
**Antidepressants other than SSRIs or SNRIs**	**N06AA, N06AF, N06AG**	**2,926**	**7.1**	**2,259**	**13.7**
Amitriptyline	N06AA09	2,562	6.2	1,877	11.4
Nortriptyline	N06AA10	1,866	4.5	88	0.5
Clomipramine	N06AA04	525	1.3	44	0.3
Others	-	542	1.3	272	1.7

The number of pregnancies prescribed individual antidepressants added up to more than number of pregnancies since some women redeemed two or more antidepressants.

© 2023 American Medical Association. All rights reserved.

eTable 5. Classes of Psycholeptic Medications Used Postpartum in Denmark (n = 2227) and Norway (n =1128)

Psychotropic medications	ATC code	Denmark		Norway	
		N of pregnancies	%	N of pregnancies	%
Oxazepam	N05BA04	506	22.7	287	25.4
Zopiclone	N05CF01	365	16.4	254	22.5
Zolpidem	N05CF02	222	10.0	52	4.6
Alprazolam	N05BA12	215	9.7	<5	<0.4
Quetiapine	N05AH04	203	9.1	99	8.8
Diazepam	N05BA01	159	7.4	137	12.1
Chlorprothixene	N05AF03	99	4.5	32	2.8
Melatonin	N05CH01	67	3.0	106	9.4
Risperidone	N05AX08	60	2.7	5	0.4
Flupentixol	N05AF01	57	2.6	5	0.4
Olanzapine	N05AH03	42	1.9	20	1.8
Hydroxyzine	N05BB01	28	1.3	62	5.5
Bromazepam	N05BA08	27	1.2	0	0.0
Lithium	N05AN01	23	1.0	7	0.6
Lorazepam	N05BA06	19	0.9	0	0.0
Levomepromazine	N05AA02	19	0.9	13	1.2
Triazolam	N05CD05	17	0.8	0	0.0
Aripiprazole	N05AX12	16	0.7	5	0.4
perphenazine	N05AB03	13	0.6	<5	<0.4
Zuclopenthixol	N05AF05	11	0.5	<5	<0.4
Prochlorperazine, melperone, ziprasidone, pimozide, amisulpride, chlordiazepoxide, clobazam, buspirone, nitrazepam, lormetazepam,temazepam, brotizolam, and zaleplon	N05AB04, N05AD03, N05AE04, N05AG02, N05AL05, N05BA02, N05BA09, N05BE01, N05CD02, N05CD06, N05CD07, N05CD09, and N05CF03	59	2.6	36	3.2

© 2023 American Medical Association. All rights reserved.

eTable 6. Associations Between Antidepressant Treatment During Pregnancy and Maternal Mental Health Outcomes in the Postpartum Year in 41 475 Pregnancies in Denmark and 16 459 Pregnancies in Norway

**Health outcomes in the postpartum**	**Denmark**					**Norway**				
	**No**	**Cases**	**IR per 1000 person years**	**Unadjusted HR (95% CI)**	**Weighted HR (95% CI)** [a]	**No**	**Cases**	**IR per 1000 person years**	**Unadjusted HR (95% CI)**	**Weighted HR (95% CI)** [a]
**Psychiatric Emergency**										
Early discontinuers	12,983	264	20.6	0.68 (0.58–0.79)	0.71 (0.60–0.84)	5,003	159	32.4	0.57 (0.47–0.70)	0.77 (0.61–0.98)
Late discontinuers (previously stable users)	8,920	269	30.8	1.01 (0.86–1.18)	1.09 (0.92–1.28)	4,583	235	52.8	0.93 (0.77–1.12)	1.00 (0.83–1.21)
Late discontinuers (short-term users)	6,599	156	23.9	0.79 (0.65–0.95)	0.69 (0.54–0.90)	3,027	100	33.7	0.60 (0.47–0.76)	0.72 (0.53–0.98)
Continuers	12,973	389	30.5	1 (ref)	1 (ref)	3,846	211	56.7	1 (ref)	1 (ref)
**Self-harm**										
Early discontinuers	12,983	35	2.7	0.87 (0.56–1.38)	0.69 (0.42–1.14)	N/A	N/A	N/A	N/A	N/A
Late discontinuers (previously stable users)	8,920	34	3.8	1.24 (0.78–1.95)	1.04 (0.65–1.68)	N/A	N/A	N/A	N/A	N/A
Late discontinuers (short-term users)	6,599	17	2.6	0.84 (0.47–1.47)	0.54 (0.28–1.06)	N/A	N/A	N/A	N/A	N/A
Continuers	12,973	40	3.1	1 (ref)	1 (ref)	N/A	N/A	N/A	N/A	N/A
**Psycholeptic medications** [b]										
Early discontinuers	12,413	623	51.5	0.79 (0.71–0.88)	0.77 (0.67–0.88)	3,600	310	89.7	0.77 (0.66–0.91)	0.85 (0.71–1.03)
Late discontinuers (previously stable users)	8,296	560	70.1	1.07 (0.96–1.20)	1.13 (1.01–1.27)	3,134	369	125.3	1.07 (0.92–1.26)	1.13 (0.97–1.33)
Late discontinuers (short-term users)	6,205	307	50.8	0.78 (0.68–0.89)	0.73 (0.61–0.87)	2,029	176	90.4	0.79 (0.64–0.94)	0.83 (0.68–1.02)
Continuers	11,640	737	65.4	1 (ref)	1 (ref)	2,487	273	116.1	1 (ref)	1 (ref)

[a] Inverse probability weight-adjusted hazard ratio.
[b] Analysis was limited to 38,554 (93.0%) pregnancies in Denmark and 11,250 (68.4%) pregnancies in Norway with no psycholeptic medication use from 6 months before pregnancy until delivery.

© 2023 American Medical Association. All rights reserved.

eTable 7. Associations Between Antidepressant Treatment During Pregnancy and Maternal Mental Health Outcomes in the Postpartum Year in Norway: Analysis Limited to the Population Having a Diagnosis of Affective Disorder in the 1 Year Before Pregnancy (14 726 in Denmark and 8542 in Norway)

Health outcomes in the postpartum	Denmark				Norway				Combined weighted HR (95% CI) [b]
	No	Cases	IR per 1000 person years	Weighted HR (95% CI) [a]	No	Cases	IR per 1000 person years	Weighted HR (95% CI) [a]	
**Psychiatric Emergency**									
Early discontinuers	3,609	130	36.8	0.75 (0.60–0.95)	2,185	111	49.8	0.74 (0.57–0.97)	0.75 (0.63–0.89)
Late discontinuers (previously stable users)	3,207	160	51.2	1.16 (0.94–1.43)	2,293	159	67.4	0.90 (0.72–1,12)	1.02 (0.80–1.31)
Late discontinuers (short-term users)	1,824	87	49.1	0.80 (0.56–1.13)	1,305	80	60.0	0.63 (0.44–0.90)	0.71 (0.55–0.91)
Continuers	6,086	264	44.5	1 (ref)	2,237	172	74.6	1 (ref)	1 (ref)
**Self-harm**									
Early discontinuers	3,609	19	5.3	0.85 (0.45–1.60)	N/A	N/A	N/A	N/A	N/A
Late discontinuers (previously stable users)	3,207	24	7.5	1.30 (0.73–2.33)	N/A	N/A	N/A	N/A	N/A
Late discontinuers (short-term users)	1,824	8	4.4	0.63 (0.26–1.51)	N/A	N/A	N/A	N/A	N/A
Continuers	6,086	25	4.1	1 (ref)	N/A	N/A	N/A	N/A	N/A
**Psycholeptic medications** [c]									
Early discontinuers	3,361	227	70.2	0.83 (0.69–1.01)	1374	152	104.4	0.95 (0.74–1.21)	0.87 (0.75–1.02)
Late discontinuers (previously stable users)	2,872	239	87.1	1.22 (1.03–1.45)	1343	227	156.9	1.37 (1.12–1.68)	1.28 (1.12–1.46)
Late discontinuers (short-term users)	1,647	115	72.6	0.93 (0.74–1.17)	730	87	112.3	0.97 (0.74–1.26)	0.95 (0.80–1.13)
Continuers	5,273	379	74.7	1 (ref)	1289	167	121.8	1 (ref)	1 (ref)

[a] Inverse probability weight-adjusted hazard ratio.
[b] Weights are from random effect analysis
[c] Analysis was limited to 13,153 (89.3%) women in Denmark and 5,369 (62.9%) women in Norway with no psycholeptic medication use from 6 months before pregnancy until delivery.

© 2023 American Medical Association. All rights reserved.

eTable 8. Associations Between Antidepressant Treatment During Pregnancy and Maternal Mental Health Outcomes in the Postpartum Year in Norway: Analysis Limited to the Population With a Diagnosis of Major Depression in the 1 Year Before Pregnancy (8617 in Denmark and 4810 in Norway)

Health outcomes in the postpartum	Denmark				Norway				Combined weighted HR (95% CI) [b]
	No	Cases	IR per 1000 person years	Weighted HR (95% CI) [a]	No	Cases	IR per 1000 person years	Weighted HR (95% CI) [a]	
**Psychiatric Emergency**									
Early discontinuers	1,973	75	39.0	0.70 (0.52–0.95)	1141	57	49.0	0.73 (0.51–1.05)	0.71 (0.52–0.95)
Late discontinuers (previously stable users)	1,852	92	51.3	1.10 (0.84–1.44)	1278	88	67.0	0.87 (0.65–1.17)	0.99 (0.78–1.24)
Late discontinuers (short-term users)	945	49	53.4	0.80 (0.56–1.14)	875	64	71.2	1.17 (0.81–1.67)	0.97 (0.67–1.40)
Continuers	3,847	175	46.7	1 (ref)	1211	96	76.8	1 (ref)	1 (ref)
**Psycholeptic medications** [c]									
Early discontinuers	1,827	115	65.4	0.77 (0.60–1.00)	722	78	102.2	1.03 (0.74–1.45)	0.87 (0.66–1.16)
Late discontinuers (previously stable users)	1,659	136	85.7	1.22 (0.98–1.52)	726	115	146.8	1.48 (1.11–1.97)	1.31 (1.09–1.58)
Late discontinuers (short-term users)	853	52	62.9	0.80 (0.58–1.11)	530	64	113.8	1.11 (0.79–1.56)	0.94 (0.68–1.29)
Continuers	3,324	241	75.3	1 (ref)	707	82	110.0	1 (ref)	1 (ref)

According to Danish legislation, the differences in the number of cases should not be less than 5 between tables. For this reason, the hazard ratios of self-harm in the postpartum period were not presented for this subgroup analysis.

[a] Inverse probability weight-adjusted hazard ratio.

[b] Weights are from random effect analysis

[c] Analysis was limited to 7,663 (88.9%) women in Denmark and 3,024 (62.9%) women in Norway with no psycholeptic medication use from 6 months before pregnancy until delivery.

© 2023 American Medical Association. All rights reserved.

eTable 9. Associations Between Antidepressant Treatment During Pregnancy and Maternal Mental Health Outcomes in the Postpartum Year in 35 463 Eligible Pregnancies in Denmark and 15 019 Eligible Pregnancies in Norway

	Denmark				Norway				Combined weighted HR (95% CI) [b]
**Health outcomes in the postpartum**	**No**	**Cases**	**IR per 1000 person years**	**Weighted HR (95% CI)** [a]	**No**	**Cases**	**IR per 1000 person years**	**Weighted HR (95% CI)** [a]	
**Psychiatric Emergency**									
Early discontinuers	11,960	242	20.5	0.69 (0.57–0.84)	4,591	144	32.0	0.75 (0.59–0.96)	0.71 (0.61–0.83)
Late discontinuers (previously stable users)	7,881	242	31.1	1.06 (0.89–1.27)	4,295	209	50.0	0.97 (0.79–1.19)	1.02 (0.89–1.17)
Late discontinuers (short-term users)	5,998	142	23.9	0.71 (0.54–0.93)	2,827	93	33.6	0.72 (0.53–1.00)	0.71 (0.58–0.88)
Continuers	9,624	299	31.6	1 (ref)	3,306	181	56.6	1 (ref)	1 (ref)
**Psycholeptic medications** [c]									
Early discontinuers	11,446	560	50.2	0.78 (0.67–0.91)	3,306	277	87.1	0.80 (0.66–0.98)	0.79 (0.70–0.89)
Late discontinuers (previously stable users)	7,350	482	68.0	1.12 (0.98–1.28)	2,950	334	120.2	1.06 (0.90–1.26)	1.10 (0.99–1.22)
Late discontinuers (short-term users)	5,643	265	48.1	0.74 (0.61–0.89)	1,898	156	85.3	0.75 (0.60–0.93)	0.74 (0.65–0.86)
Continuers	8,595	510	61.2	1 (ref)	2,086	235	119.3	1 (ref)	1 (ref)

According to Danish legislation, the differences in the number of cases should not be less than 5 between tables. For this reason, the hazard ratios of self-harm in the postpartum period were not presented for this subgroup analysis.

[a] Inverse probability weight-adjusted hazard ratio.

[b] Weights are from random effect analysis

[c] Analysis was limited to 33,034 (93.2%) women in the Danish cohort and 10,240 (68.2%) women in the Norwegian cohort with no psycholeptics medication use from 6 months before pregnancy until delivery.

© 2023 American Medical Association. All rights reserved.

eTable 10. Associations Between Antidepressant Treatment During Pregnancy and Maternal Mental Health Outcomes in the Postpartum Year in Denmark Among Term Pregnancies (38 267 in Denmark and 15 328 in Norway)

**Health outcomes in the postpartum**	**Denmark**				**Norway**				**Combined weighted HR (95% CI)** [b]
	**No**	**Cases**	**IR per 1000 person years**	**Weighted HR (95% CI)** [a]	**No**	**Cases**	**IR per 1000 person years**	**Weighted HR (95% CI)** [a]	
**Psychiatric Emergency**									
Early discontinuers	12,151	252	21.0	0.75 (0.63–0.91)	4,551	148	33.2	0.79 (0.62–1.00)	0.76 (0.66–0.88)
Late discontinuers (previously stable users)	8,374	245	29.8	1.08 (0.91–1.29)	4,393	227	53.2	1.01 (0.92–1.23)	1.04 (0.83–1.16)
Late discontinuers (short-term users)	6,143	143	23.6	0.69 (0.53–0.90)	2,835	98	35.3	0.71 (0.51–0.98)	0.70 (0.57–0.86)
Continuers	11,599	339	29.7	1 (ref)	3,549	194	56.5	1 (ref)	1 (ref)
**Psycholeptic medications** [c]									
Early discontinuers	11,625	580	51.2	0.78 (0.68–0.90)	3,287	276	87.4	0.83 (0.69–1.01)	0.80 (0.71–0.89)
Late discontinuers (previously stable users)	7,808	516	68.5	1.11 (0.99–1.25)	3,036	348	121.7	1.11 (0.94–1.31)	1.11 (1.01–1.22)
Late discontinuers (short-term users)	5,782	283	50.2	0.72 (0.60–0.87)	1,898	159	87.2	0.80 (0.65–0.98)	0.75 (0.66–0.87)
Continuers	10,471	661	65.2	1 (ref)	2,307	250	114.5	1 (ref)	1 (ref)

According to Danish legislation, the differences in the number of cases should not be less than 5 between tables. For this reason, the hazard ratios of self-harm in the postpartum period were not presented for this subgroup analysis.

[a] Inverse probability weight-adjusted hazard ratio.

[b] Weights are from random effect analysis

[c] Analysis was limited to 35,686 (93.3%) women in Denmark and 10,528 (68.7%) women in Norway with no psycholeptic medication use from 6 months before pregnancy until delivery.

© 2023 American Medical Association. All rights reserved.

eTable 11. Associations Between Antidepressant Treatment During Pregnancy and Initiation of Psycholeptic Medications, Excluding Melatonin, in the Postpartum Year in 38 587 Pregnancies in Denmark and 11 588 Pregnancies in Norway With No Psycholeptic Use From 6 Months Before Pregnancy Until Delivery

**Health outcomes in the postpartum**	**Denmark**				**Norway**				**Combined weighted HR (95% CI)** [b]
	**No**	**Cases**	**IR per 1000 person years**	**Weighted HR (95% CI)** [a]	**No**	**Cases**	**IR per 1000 person years**	**Weighted HR (95% CI)** [a]	
**Psycholeptic medications** [c]									
Early discontinuers	12,423	609	50.3	0.76 (0.67–0.88)	3,705	293	82.2	0.83 (0.69–1.01)	0.78 (0.70–0.87)
Late discontinuers (previously stable users)	8,301	549	68.6	1.12 (1.00–1.26)	3,242	358	117.2	1.09 (0.93–1.28)	1.11 (1.01–1.22)
Late discontinuers (short-term users)	6,209	301	49.7	0.72 (0.60–0.86)	2,088	165	82.1	0.73 (0.60–0.88)	0.72 (0.64–0.83)
Continuers	11,654	725	64.2	1 (ref)	2,553	273	113.0	1 (ref)	1 (ref)

[a] Inverse probability weight-adjusted hazard ratio.
[b] Weights are from random effect analysis

© 2023 American Medical Association. All rights reserved.

eTable 12. Associations Between Antidepressant Treatment During Pregnancy and Maternal Mental Health Outcomes in the Postpartum Year in Denmark

Health outcomes in the postpartum	No	Cases	IR per 1000 person years	Unadjusted HR (95% CI)	Weighted HR (95% CI) [a]	Weighted HR (95% CI) [b]	Weighted HR (95% CI) [c]	Weighted HR (95% CI) [d]
**Psychiatric Emergency**								
Early discontinuers	12,983	264	20.6	0.68 (0.58–0.79)	0.71 (0.60–0.84)	0.71 (0.60–0.84)	0.70 (0.59–0.83)	0.70 (0.59–0.83)
Late discontinuers (previously stable users)	8,920	269	30.8	1.01 (0.86–1.18)	1.09 (0.92–1.28)	1.09 (0.92–1.28)	1.09 (0.92–1.28)	1.09 (0.92–1.28)
Late discontinuers (short-term users)	6,599	156	23.9	0.79 (0.65–0.95)	0.69 (0.54–0.90)	0.69 (0.54–0.90)	0.66 (0.51–0.85)	0.69 (0.53–0.89)
Continuers	12,973	389	30.5	1 (ref)	1 (ref)	1 (ref)	1 (ref)	1 (ref)
**Self-harm**								
Early discontinuers	12,983	35	2.7	0.87 (0.56–1.38)	0.69 (0.42–1.14)	0.69 (0.42–1.14)	0.69 (0.42–1.14)	0.69 (0.42–1.14)
Late discontinuers (previously stable users)	8,920	34	3.8	1.24 (0.78–1.95)	1.04 (0.65–1.68)	1.04 (0.65–1.68)	1.03 (0.64–1.65)	1.03 (0.64–1.65)
Late discontinuers (short-term users)	6,599	17	2.6	0.84 (0.47–1.47)	0.54 (0.28–1.06)	0.55 (0.28–1.06)	0.50 (0.26–0.96)	0.54 (0.27–1.07)
Continuers	12,973	40	3.1	1 (ref)	1 (ref)	1 (ref)	1 (ref)	1 (ref)
**Psycholeptic medications** [d]								
Early discontinuers	12,413	623	51.5	0.79 (0.71–0.88)	0.77 (0.67–0.88)	0.77 (0.67–0.88)	0.77 (0.67–0.88)	0.77 (0.67–0.88)
Late discontinuers (previously stable users)	8,296	560	70.1	1.07 (0.96–1.20)	1.13 (1.01–1.27)	1.13 (1.01–1.27)	1.13 (1.00–1.26)	1.13 (1.00–1.26)
Late discontinuers (short-term users)	6,205	307	50.8	0.78 (0.68–0.89)	0.73 (0.61–0.87)	0.73 (0.61–0.87)	0.70 (0.59–0.85)	0.74 (0.62–0.88)
Continuers	11,640	737	65.4	1 (ref)	1 (ref)	1 (ref)	1 (ref)	1 (ref)

[a] Inverse probability weight-adjusted hazard ratio.
[b] Further accounting for the censoring due to emigration and death, which concerned 96 (0.2%) women who emigrated or died within one year postpartum.
[c] Further adjustment for highest education attained and income in the year of pregnancy
[d] Further adjustment for imbalanced covariates between late discontinuers (short-term users) versus continuers, including depression before pregnancy, schizophrenia before pregnancy, co-prescribed benzodiazepines within 6 months before pregnancy, and calendar year of pregnancy.
[e] Analysis was limited to 38,554 (93.0%) women with no psycholeptic medication use from 6 months before pregnancy until delivery

© 2023 American Medical Association. All rights reserved.

eFigure 1. Standardized Mean Difference in Confounding Factors Before and After Inverse Probability of Treatment Weighting (IPTW) in Early Discontinuers vs Continuers

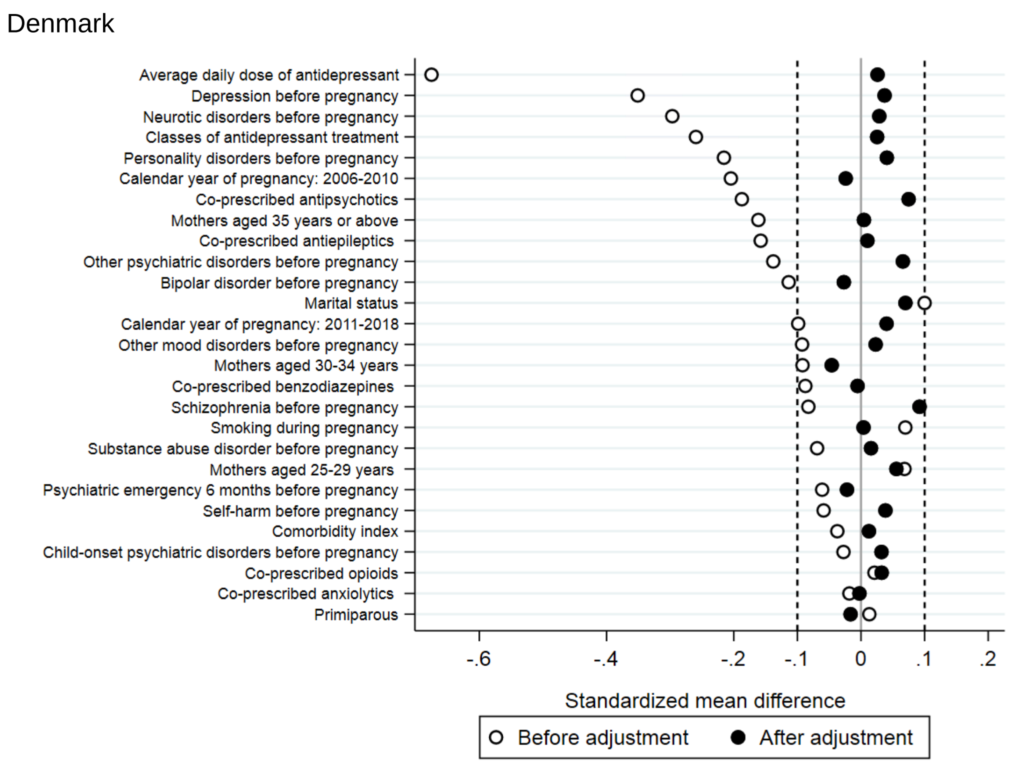

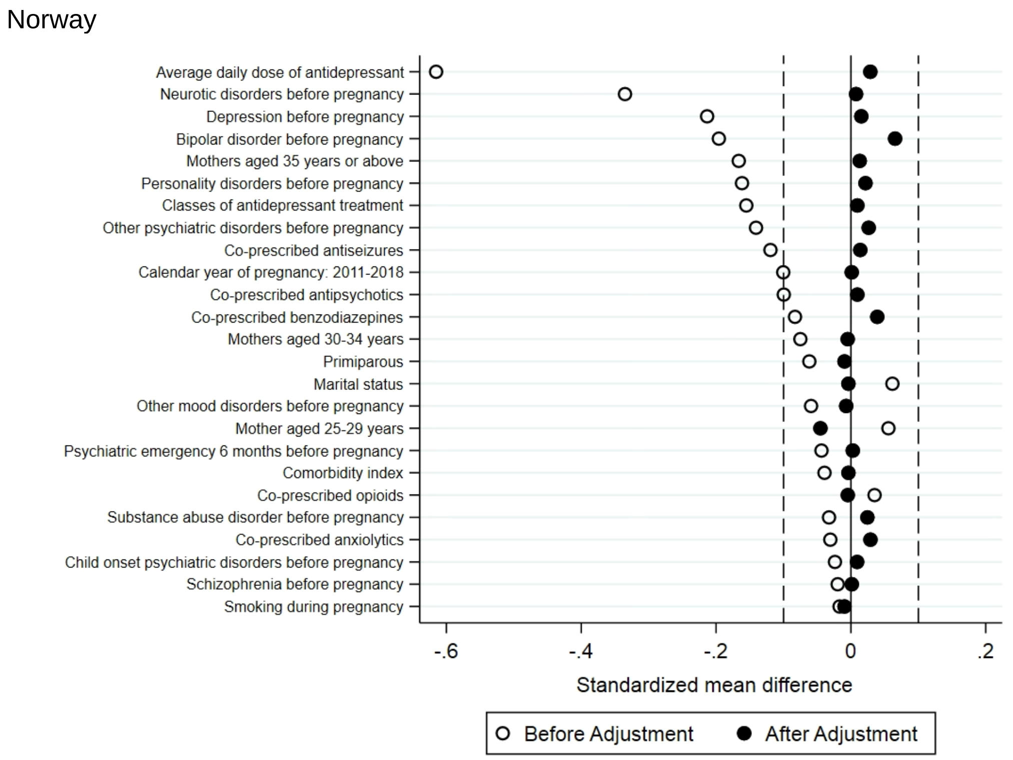

A standardized mean difference <0.1 indicates characteristics are satisfactorily balanced.

© 2023 American Medical Association. All rights reserved.

eFigure 2. Standardized Mean Difference in Confounding Factors Before and After Inverse Probability of Treatment Weighting (IPTW) in Late Discontinuers (Previously Stable Users) vs Continuers

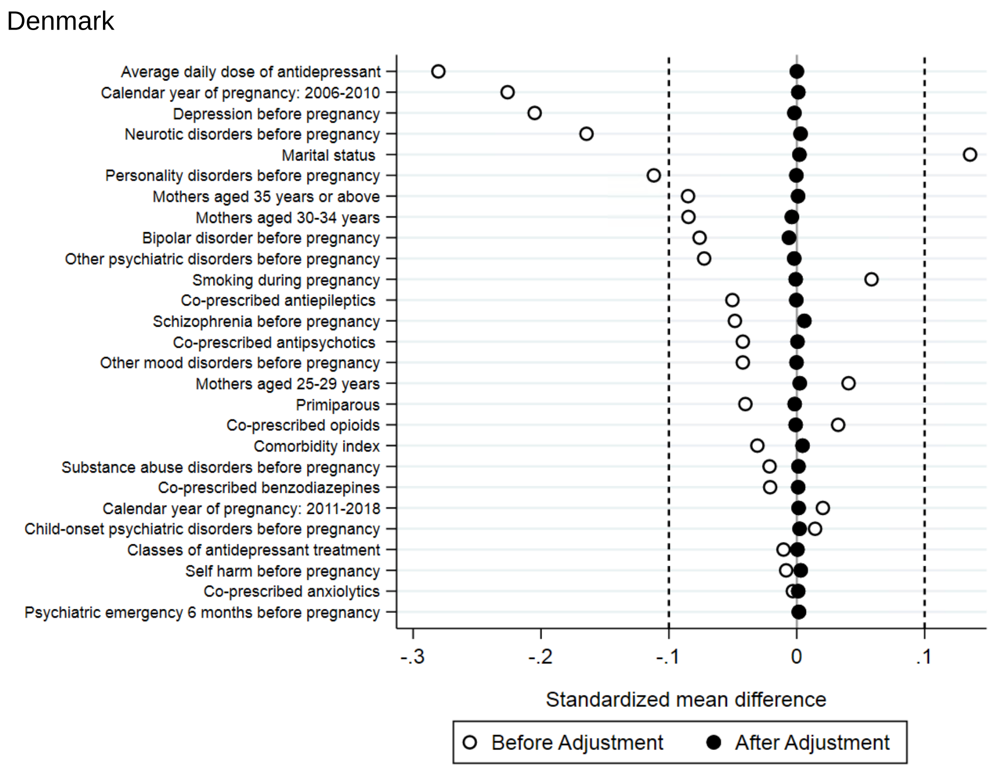

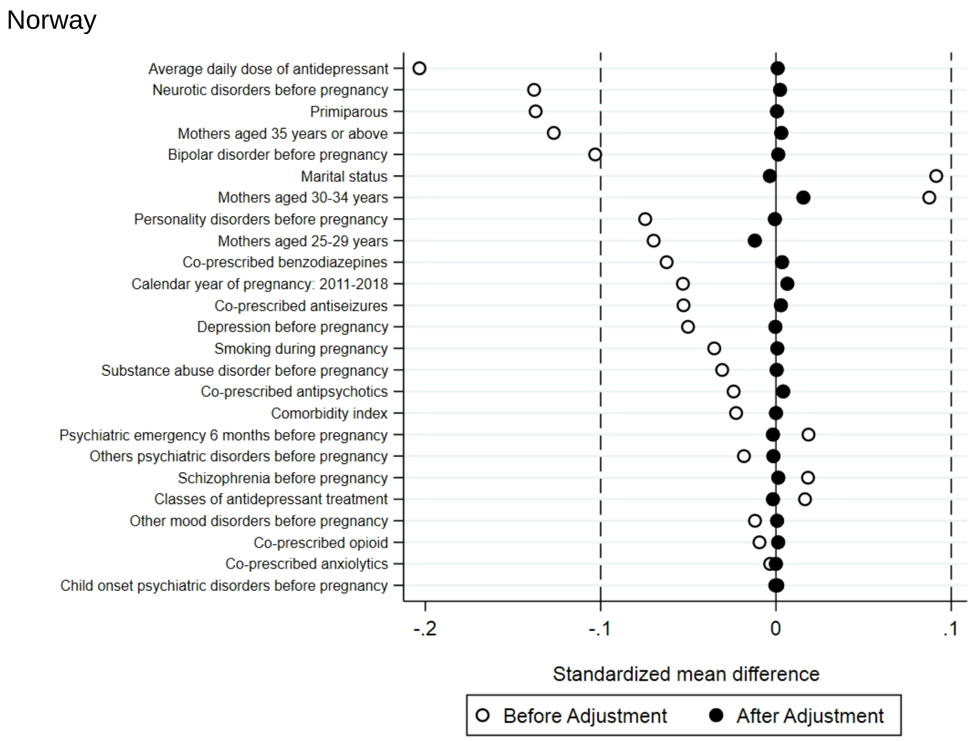

A standardized mean difference <0.1 indicates characteristics are satisfactorily balanced.

© 2023 American Medical Association. All rights reserved.

eFigure 3. Standardized Mean Difference in Confounding Factors Before and After Inverse Probability of Treatment Weighting (IPTW) in Late Discontinuers (Short-Term Users) vs Continuers

Denmark

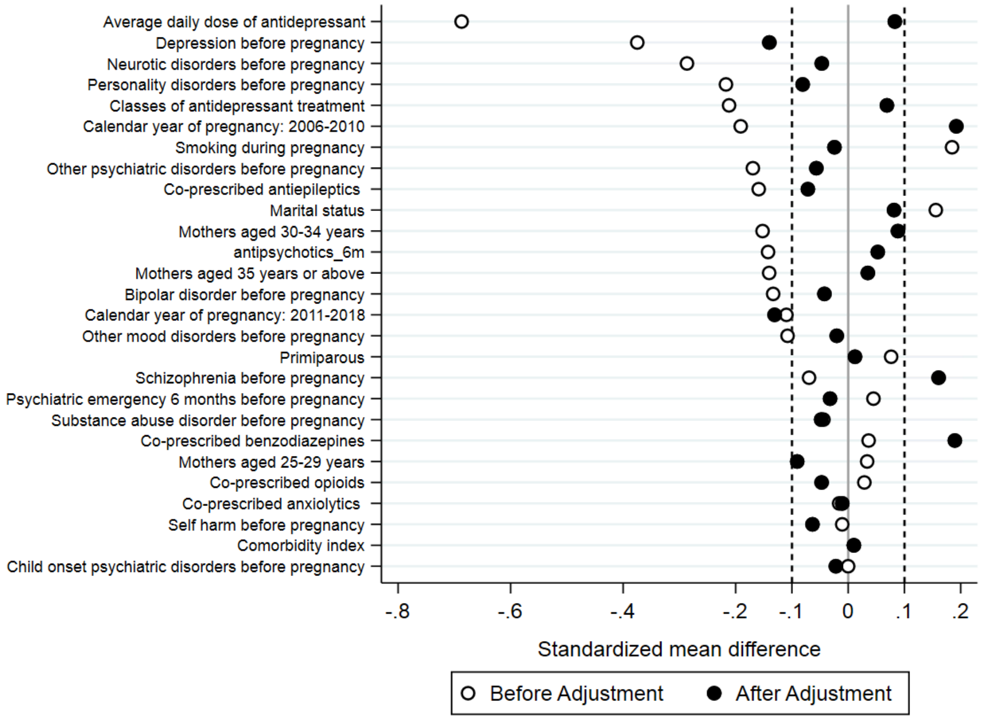

Norway

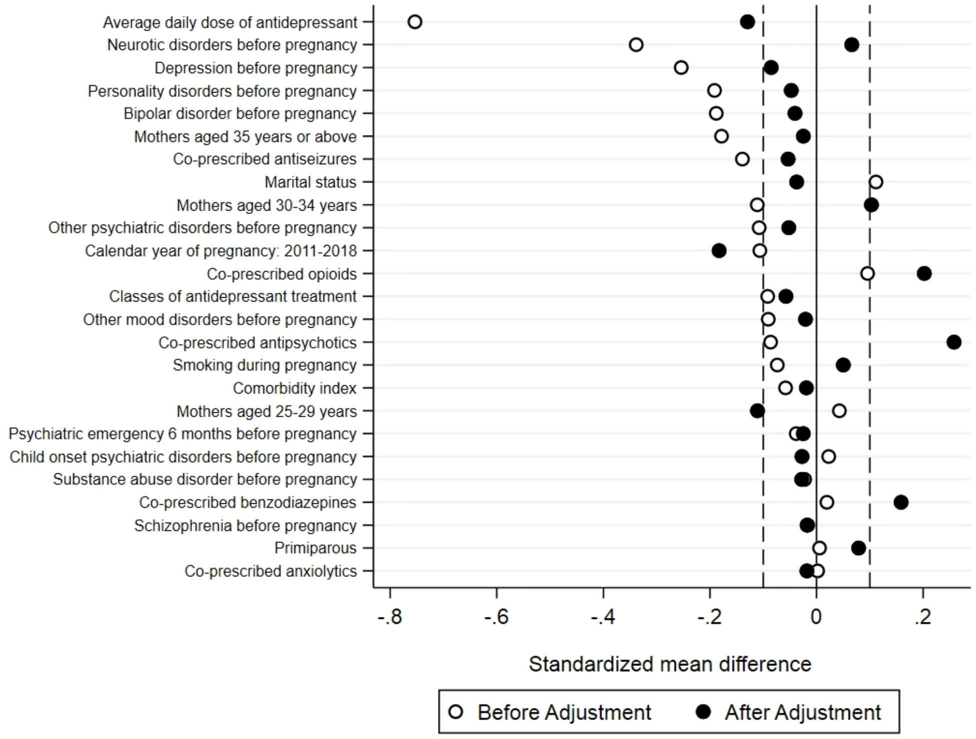

A standardized mean difference <0.1 indicates characteristics are satisfactorily balanced.

© 2023 American Medical Association. All rights reserved.

eReferences

1. Bliddal M, Broe A, Pottegård A, Olsen J, Langhoff-Roos J. The Danish Medical Birth Register. *European journal of epidemiology.* 2018;33(1):27-36.
2. Jørgensen FS. Epidemiological studies of obstetric ultrasound examinations in Denmark 1989-1990 versus 1994-1995. *Acta obstetricia et gynecologica Scandinavica.* 1999;78(4):305-309.
3. Irgens LM. The Medical Birth Registry of Norway. Epidemiological research and surveillance throughout 30 years. *Acta obstetricia et gynecologica Scandinavica.* 2000;79(6):435-439.
4. Gasse C, Danielsen AA, Pedersen MG, Pedersen CB, Mors O, Christensen J. Positive predictive value of a register-based algorithm using the Danish National Registries to identify suicidal events. *Pharmacoepidemiology and drug safety.* 2018;27(10):1131-1138.
5. Ording AG, Cronin-Fenton DP, Jacobsen JB, et al. Comorbidity and survival of Danish breast cancer patients from 2000-2011: a population-based cohort study. *Clinical epidemiology.* 2013;5(Suppl 1):39-46.

© 2023 American Medical Association. All rights reserved.
