# Supplementary material for: Timing of Antidepressant Discontinuation During Pregnancy and Postpartum Psychiatric Outcomes in Denmark and Norway
Source: JAMA Psychiatry. 2023 Mar 8;80(5):441–50. doi: 10.1001/jamapsychiatry.2023.0041 (PMC9996461; doi:10.1001/jamapsychiatry.2023.0041)
Supplement: Supplement 2. — Data Sharing Statement [file jamapsychiatry-e230041-s002.pdf]

Data Sharing Statement

Trinh. Timing of Antidepressant Discontinuation During Pregnancy and Psychiatric Outcomes Post Partum in Denmark and Norway. *JAMA Psychiatry*. Published March 08, 2023. doi:10.1001/jamapsychiatry.2023.0041

Data
Data available: No

Additional Information
Explanation for why data not available: The study was based on the nationwide registers in Denmark and Norway. According to Danish and Norwegian legislation, individual-level data can be accessed only through secure servers, Denmark Statistics and the Service for Sensitive Data – TSD at the University of Oslo in Norway, where download or export of individual-level information is prohibited. In Norway, data are available for the researchers upon request to the registry holders, provided legal and ethical approvals. In Denmark, only aggregated data can be shared to ensure complete anonymity and protection of individuals included in the studies.
